# The Use of Bayesian Latent Class Cluster Models to Classify Patterns of Cognitive Performance in Healthy Ageing

**DOI:** 10.1371/journal.pone.0071940

**Published:** 2013-08-20

**Authors:** Patrício Soares Costa, Nadine Correia Santos, Pedro Cunha, Joana Almeida Palha, Nuno Sousa

**Affiliations:** 1 Life and Health Sciences Research Institute (ICVS), School of Health Sciences, University of Minho, Braga, Portugal; 2 ICVS/3B’s, PT Government Associate Laboratory, Braga/Guimarães, Portugal; 3 Centro Hospital Alto Ave – EPE, Guimarães, Portugal; Hospital General Dr. Manuel Gea González, Mexico

## Abstract

The main focus of this study is to illustrate the applicability of latent class analysis in the assessment of cognitive performance profiles during ageing. Principal component analysis (PCA) was used to detect main cognitive dimensions (based on the neurocognitive test variables) and Bayesian latent class analysis (LCA) models (without constraints) were used to explore patterns of cognitive performance among community-dwelling older individuals. Gender, age and number of school years were explored as variables. Three cognitive dimensions were identified: general cognition (MMSE), memory (MEM) and executive (EXEC) function. Based on these, three latent classes of cognitive performance profiles (LC1 to LC3) were identified among the older adults. These classes corresponded to stronger to weaker performance patterns (LC1>LC2>LC3) across all dimensions; each latent class denoted the same hierarchy in the proportion of males, age and number of school years. Bayesian LCA provided a powerful tool to explore cognitive typologies among healthy cognitive agers.

## Introduction

Normal ageing is associated with cognitive decline in various memory and executive function abilities, the course of which varies between individuals and in the same individual over the lifespan [1]–[2]. The identification of cognitive patterns in the ageing population is a fundamental step in studies assessing the effect of intrinsic and extrinsic factors on cognitive performance. Although it is impossible to apply all neuropsychological/cognitive assessment parameters in a given population sample, different neurocognitive test variables have similar patterns and relation with age (see for review [3]). The use of composite scores and cluster analysis methods to identify test variable groupings and to provide insights into distinct groups of cognitive patterns can be particularly powerful in studying cognitive trajectories in population studies. On this, latent class analysis (LCA) cluster modeling may be especially relevant in that i) it does not conform to model assumptions (for example, normal distribution and homogeneity), ii) it can include variables of mixed scale types in the same analysis, and iii) the relationship between the latent classes and covariates can be assessed simultaneously [4]–[6]. Furthermore, the mixture model-based approach allows to estimate membership probabilities (to classify cases into the appropriate cluster) and to explore hidden clusters represented by each latent class [4]–[6].

The present work from the Switchbox Consortium (http://www.switchbox-online.eu/) is based on findings in older community-dwelling individuals (residing in Minho, Northern Portugal) that were cognitively characterized via a battery of neurocognitive tests [7]. Here, based on the neurocognitive measures, the study aim was two-fold: first, to identify neurocognitive variables’ grouping(s); second, determine the value of Bayesian LCA on modeling patterns of cognitive performance among older individuals.

## Materials and Methods

### Study Sample

Participants (n = 506) were randomly selected from the Guimarães and Vizela local area health authority registries (health care centers). All participants still lived in the community (community-dwellers), with equal distribution between urban and rural areas. The majority of individuals (70.4%, females 51.9%) were in the medium socio-economic stratum (Class III, Graffar measure [8]) and retired (n = 344, females 48.3%). The cohort was representative of the general Portuguese population with respect to gender (females, n = 264 or 52.2%), age [range: 50–89 years; M = 65.7, SD = 8.98; age categories: [50–60[, 30.2% (females, 55.6%); [60–70[, 33.2% (females, 54.8%); [70+[, 36.6% (females, 47.0%)], and education (median years of schooling = 4; 1.2, 5.9, 73.9, 7.7, 9.1, and 2.2% of the cohort attended school for 0, 1–2, 3–4, 5–8, 9–12, and 13+ years, respectively; literacy rate 99.4%, able to read and write).

The study was conducted in accordance with the Declaration of Helsinki (59^th^ Amendment) and was approved by the national ethical committee (Comissão Nacional de Protecção de Dados) and by the local ethics review boards (Hospital Escola Braga, Braga; Centro Hospitalar do Alto Ave, Guimarães; and Unidade Local de Saúde do Alto Minho, Viana-do-Castelo/Ponte-de-Lima). Potential participants were explained the study goals and the neurocognitive assessments. All volunteers provided written informed consent. The primary exclusion criteria included inability to understand informed consent, participant choice to withdraw from the study, incapacity and/or inability to attend the neuropsychological assessment session(s), and/or diagnosed dementia or neuropsychiatric disorder. Further exclusion/inclusion selection criteria are described elsewhere [7], [9].

### Neurocognitive Evaluation

Tests were selected to provide cognitive profiles (general cognition, executive and memory functions), as previously reported [7], [9]. The following parameters were assessed: global cognitive status with the mini-mental state examination (MMSE) [10]; short-term verbal memory with the digit span forward test (subtest of the Wechsler adult intelligence test WAIS III, 1997) [11], verbal working memory with the digit span backward test (subtest of the Wechsler adult intelligence test WAIS III, 1997) [11] and multiple trial verbal learning and memory with the selective reminding test [SRT, parameters: consistent long term retrieval (CLTR), long term storage (LTS), delayed recall and intrusions] [12]; response inhibition/cognitive flexibility with the Stroop color and word test (Stroop, parameters: words, colors and words/colors) [13] and verbal fluency with the controlled oral word association test F-A-S (COWAT-FAS, parameters: admissible and non-admissible) [14]. A team of trained psychologists conducted the assessments.

### Analysis Methodology

Data analysis followed previously reported strategies [7] and was structured as follows:

1. Conversion of all test scores into z scores to express all variables in the same scale;
2. Exclusion of participants that met the previously established MMSE criteria for cognitive impairment: total MMSE score <17 if individual with less than 4 years of formal school education and/or ≥72 years of age (excluded n = 1 individual), or total MMSE score <23 if individual with more than 4 years of formal school education and/or ≤71 years of age (excluded n = 2 individuals) [7], [9];
3. Principal component analysis (PCA) for allocation of multiple test parameters into single or composite cognitive dimensions, allowing to reduce information of multiple parameters into a minimal number of components (dimensions) and the identification of the “weight” of each component (ultimately also allowing to calculate the performance score of each individual in each dimension);
4. Bayesian latent class analysis (LCA) cluster modeling for determination of cognitive performance patterns;
5. ANOVA, MANOVAS, t-tests and chi-squared tests to detect differences between the subjects in the distinct latent classes for relevant variables.

The SPSS package v20 (IBM SPSS Statistics) and AMOS statistical package v21 [15] were used to conduct all statistical analysis. R and R Commander was used to generate scatter and 3D plots [16], [17].

### Principal Component Analysis

The following measures (neurocognitive tests’ total or parameter scores) were considered: MMSE, Stroop, SRT, COWAT F-A-S and digits. The analysis (extraction method: principal component analysis; rotation method: Varimax with Kaiser Normalization) included all individuals that met the previously established MMSE threshold [7], [9] and had no missing values in any of the considered neurocognitive measures (n = 503). Here PCA was used so to maintain the largest possible total explained variance as opposed to common variance.

### Bayesian Latent Class Analysis

Bayesian LCA cluster modeling with Markov chain Monte Carlo (MCMC) simulation technique was used to identify latent classes (LC) that grouped together individuals similar in cognitive performance (in respect to the identified PCA cognitive dimensions). The components (cognitive dimensions) were stipulated as uncorrelated and no pre-established group coding was defined regarding cognitive performance (to account for the possibility of label switching [18], trace plots were run for the individual parameters). Latent structure analysis is a variation of mixture modeling in which the measured variables are required to be modeled as independent within each group. As such, an essential assumption of LCA is that of “conditional independence”, where, when the measured variables are multivariate normal, they are required to be modeled as uncorrelated [15]. Finally, Bayesian LCA allows for a more sophisticated group division (proportion estimates) than, for example, a quantile approach, by not forcing similar group sample sizes across divisions.

Four separate Bayesian LCA solutions were tested (consisting of 2 to 5 clusters, respectively; seed used in all solutions = 2,992,372; default burn-in period = 500) and all solutions satisfied the Gelman et al. [4] default convergence criteria of <1.10 (the AMOS default criterion of 1.002 is considered conservative). Cluster model solutions were stopped at a number of samples of approximately 55,500, yielding for each a overall convergence statistic (CS, as it approaches 1.000 there is no much more precision to be gained) of: 2-cluster, CS = 1.0003; 3-cluster, CS = 1.0003; 4-cluster, CS = 1.0248; 5-cluster, CS = 1.0257 [this solution was not further considered as it yielded two latent classes that contained a proportion estimate of 0.038 and 0.092, respectively, and CS was not reached for three of the latent classes, for the number of samples considered]. For each model (cluster solution), the probabilities of membership in each latent class group were inputted into the dataset based on the highest probability value. In addition to the CS value, a frequency polygon of the distribution of the proportion was also used to check convergence of the Bayesian MCMC method (in frequency polygons, when the distributions of the first and last thirds of the analysis samples are virtually identical/overlap then the posterior distribution has converged; in addition, the center of the distribution represents the mean value of the parameter and the standard deviation can also be visualized) (Fig. S1).

### Scatterplot Matrix and 3D Scatterplots

R and R Commander were used to perform a scatterplot matrix by LC (representing the least-squares lines, smooth lines and spread; density plots on diagonal) for the three cognitive dimensions explored and a 3D scatterplot with the cognitive dimension zMMSE as response variable, and the dimensions zMEM and zEXEC as explanatory variables (options: show axis scales; show surface grid lines; smooth regression; plot 50% concentration ellipsoid, plot by LC and parallel regression surfaces).

## Results

### Cognitive Performance Dimensions

From the PCA analysis three dimensions were identified: one single (termed “*MMSE”*) and two composites (termed “*EXEC”* and “*MEM”*) (sampling adequacy, KMO = 0.838; Bartlett’s sphericity, χ^2^_(66)_ = 2145.6, p<0.001, revealing significant correlation between the variables) (Table 1). The parameter MMSE, a general cognition measure, was considered a single dimension (*MMSE*) because it loaded in 2 components with similar weights (loadings less than 0.5, and the difference between the two components’ weights was of less than 0.1). The composite dimensions were formed as follows: executive function (*EXEC*, Cronbach’s alpha 0.780) comprising Stroop measures (parameters: words, colors and words/colors), COWAT F-A-S (parameter: admissible) and digits (parameters: forward and backward spans); and, memory function (*MEM*, Cronbach’s alpha 0.894) composed of the SRT test variables (parameters: CLTR, LTS and delayed recall). The dimensional composite consisting of the parameters SRT intrusions and FAS non-admissible (which would reflect test “errors”) was not considered viable due to the very low Cronbach’s alpha (0.000). Results were confirmed using oblimin rotation, meaning that the factorial structural remained the same independently of the rotation method.

**Table 1:** Principal component analysis with Varimax rotation with Kaiser normalization.

	Communalities	Component		
		1	2	3
zStroop words	0.663	0.791	0.154	−0.119
zStroop_colors	0.640	0.736	0.215	−0.230
zCOWAT F-A-S admissible	0.539	0.666	0.246	0.187
zStroop words/colors	0.543	0.603	0.051	−0.421
zDigits forward	0.464	0.587	0.059	0.340
zDigits backward	0.472	0.569	0.369	0.107
zMMSE	0.403	0.484	0.411	−0.003
zSRT LTS	0.836	0.218	0.884	−0.077
zSRT CLTR	0.825	0.187	0.883	−0.097
zSRT delayed recall	0.753	0.226	0.836	−0.056
ZSRT intrusions	0.451	0.011	0.065	0.668
zCOWAT F-A-S non-admissible	0.246	−0.005	−0.192	0.458
Eigenvalue		3.009	2.744	1.081
% of Variance (Cumulative %)		25.1	22.9(47.9)	9.0 (56.9)
Cronbach’s Alpha		0.780	0.894	0.000

### Latent Classes of Cognitive Performance

**Figure 1:**
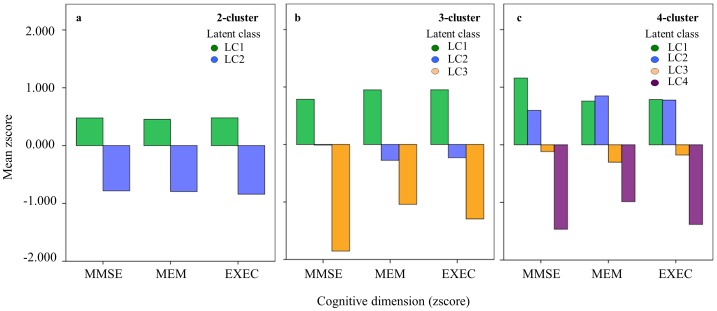
Latent classes performance in each cognitive dimension for each Bayesian LCA solution. **a** 2-cluster (latent classes, LC1 to LC2), **b** 3-cluster (LC1 to LC3), **c** 4-cluster (LC1 to LC4).

**Figure 2:**
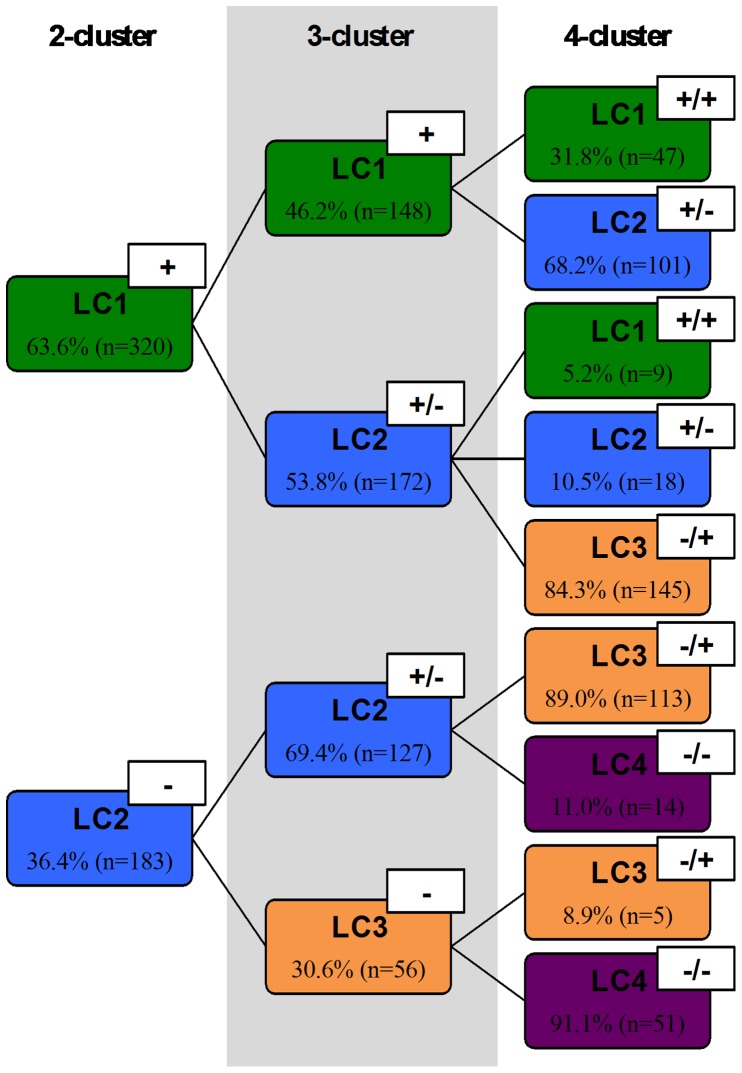
Schematic representation of the latent class membership distribution across the 2-, 3- and 4-cluster LCA solutions based on cross tabulation analysis. Frequency counts and percentages of subjects are reported for each latent class in each cluster solution.

Based on the PCA dimensions multiple Bayesian LCA clustering solutions were tested (2-, 3- and 4-cluster) (Table 2 and Fig. S1). The general effect size η^2^ was derived by dividing the sum of all between- groups sum of squares by the sum of the total sum of groups; for the 2-, 3-, 4-cluster solutions, η^2^ was 0.386, 0.506 and 0.478, respectively. The latent classes in each of the Bayesian LCA clustering solutions represented distinct cognitive typologies (Fig. 1); cross-tabulation analysis allowed to determine about the membership interrelation of the cluster solutions and to determine how subjects were divided as the number of cluster solutions increased (Fig. 2). For the 2-cluster solution two separate latent classes were identified: LC1 and LC2, grouping, respectively, stronger (+) and weaker (-) performers. In the next tested solution (3-cluster), the identified latent classes subdivided the cohort into “stronger” (+, LC1), “average” (+/−, LC2) and “weaker” (−, LC3) performers. Cross tabulation analysis showed that the “average” latent class resulted both from subjects from the “stronger” and “weaker” latent classes identified in the 2-cluster model. Finally, the 4-cluster solution further subdivided the sample into different cognitive performance patterns, from “stronger” (+/+, LC1), to “average high” (+/−, LC2), “average low” (−/+, LC3) and “weaker” (−/−, LC4). The first two classes (LC1 and LC2) resulted from differentiation among the LC1 and LC2 3-cluster latent classes, while the latter two (LC3 and LC4) from the LC2 and LC3 3-cluster latent classes. In all cluster solutions, subjects within each latent class performed similarly across the cognitive dimensions (that is, a performance pattern in a particular dimension corresponded to a similar “stronger”, “high/low average” or “weaker” performance in the other dimensions) (Fig. 1).

**Table 2:** Results and latent classes distribution for each Bayesian LCA cluster solution.

	2-cluster		3-cluster			4-cluster			
	LC1	LC2	LC1	LC2	LC3	LC1	LC2	LC3	LC4
Proportion	0.630	0.370	0.305	0.550	0.144	0.115	0.271	0.478	0.136
SE	0.001	0.001	0.001	0.001	0.001	0.000	0.002	0.002	0.001
SD	0.041	0.041	0.040	0.041	0.028	0.014	0.029	0.032	0.018
CS	1.000	1.000	1.000	1.000	1.000	1.000	1.004	1.002	1.001
95% Lower bound	0.550	0.292	0.229	0.467	0.096	0.088	0.216	0.416	0.102
95% Upper bound	0.708	0.450	0.386	0.630	0.204	0.144	0.329	0.540	0.174
Skewness	−0.066	0.066	0.089	−0.090	0.423	0.197	0.331	−0.066	0.173
Kurtosis	−0.045	−0.045	−0.034	−0.003	0.324	0.107	1.092	0.241	0.030
Min	0.463	0.211	0.149	0.356	0.056	0.058	0.167	0.286	0.066
Max	0.789	0.537	0.482	0.697	0.291	0.181	0.492	0.606	0.216
n	320	183	148	299	56	56	119	263	65
%	63.6%	36.4%	29.4%	59.4%	11.1%	11.1%	23.7%	52.3%	12.9%

LC = latent class; SE = standard error; SD = standard deviation; CS = convergence statistic.

To explore basic participant socio-demographic characteristics and how these might underlie cognitive performance, the variables gender, age and school years were used to describe and compare between subjects in the latent classes of each Bayesian LCA solution (Table 3). Among others, these factors were identified as relevant in previous studies [7], [9]. As expected, with exception of gender in the 2- and 4-cluster models, all factors were significant in explaining latent class membership; females, older subjects and those with less school years belonged to latent classes denoting weaker cognitive performance across the studied cognitive dimensions.

**Table 3:** Latent classes characterization for each Bayesian LCA cluster solution.

		2-cluster		3-cluster			4-cluster			
		LC1	LC2	LC1	LC2	LC3	LC1	LC2	LC3	LC4
Gender, %	Male	50.6	43.2	52.7	48.5	32.1#	53.6	51.3	48.7	33.8
	Female	49.4	56.8	47.3	51.5	67.9#	46.4	48.7	51.3	66.2
		?^2^_(1)_ = 2.593, p = 0.107		?^2^_(2)_ = 6.982, p = 0.030			?^2^_(3)_ = 6.467, p = 0.091			
Age, mean (SD)		63.1 (8.37)	70.2 (8.13)	60.9 (3.49)^c^	66.9 (8.43)^b^	71.4 (8.71)^a^	62.0 (8.38)^c^	61.7 (8.10)^c^	66.6 (8.56)^b^	72.3 (7.61)^a^
		t_(501)_ = −9.29, p<0.001		F_(2,500)_ = 40.7, p<0.001			F_(3,499)_ = 27.3, p<0.001			
		Cohen’s d = −0.83		η^2^ = 0.14			η^2^ = 0.14			
School years, mean (SD)		5.2 (2.92)	3.7 (1.99)	6.2 (3.49)^a^	4.2 (2.06)^b^	3.1 (1.05)^c^	6.2 (3.86)^a^	5.8 (3.12)^a^	4.2 (2.08)^b^	3.1 (1.23)^c^
		t_(484.4)_ = −6.56, p<0.001		F_Welch(2,144.7)_ = 41.4, p<0.001			F_Welch(3,159.2)_ = 30.1, p<0.001			
		Cohen’s d = −0.60		η^2^ = 0.16			η^2^ = 0.13			

# Adjusted standardized residual above |1.96|, more females in LC3; ^a,b,c^ Different letters represent significant differences (post hoc Bonferroni for equal variances assumed and Games-Howell for equal variances not assumed). LC = latent class; SD = standard deviation.

**Figure 3:**
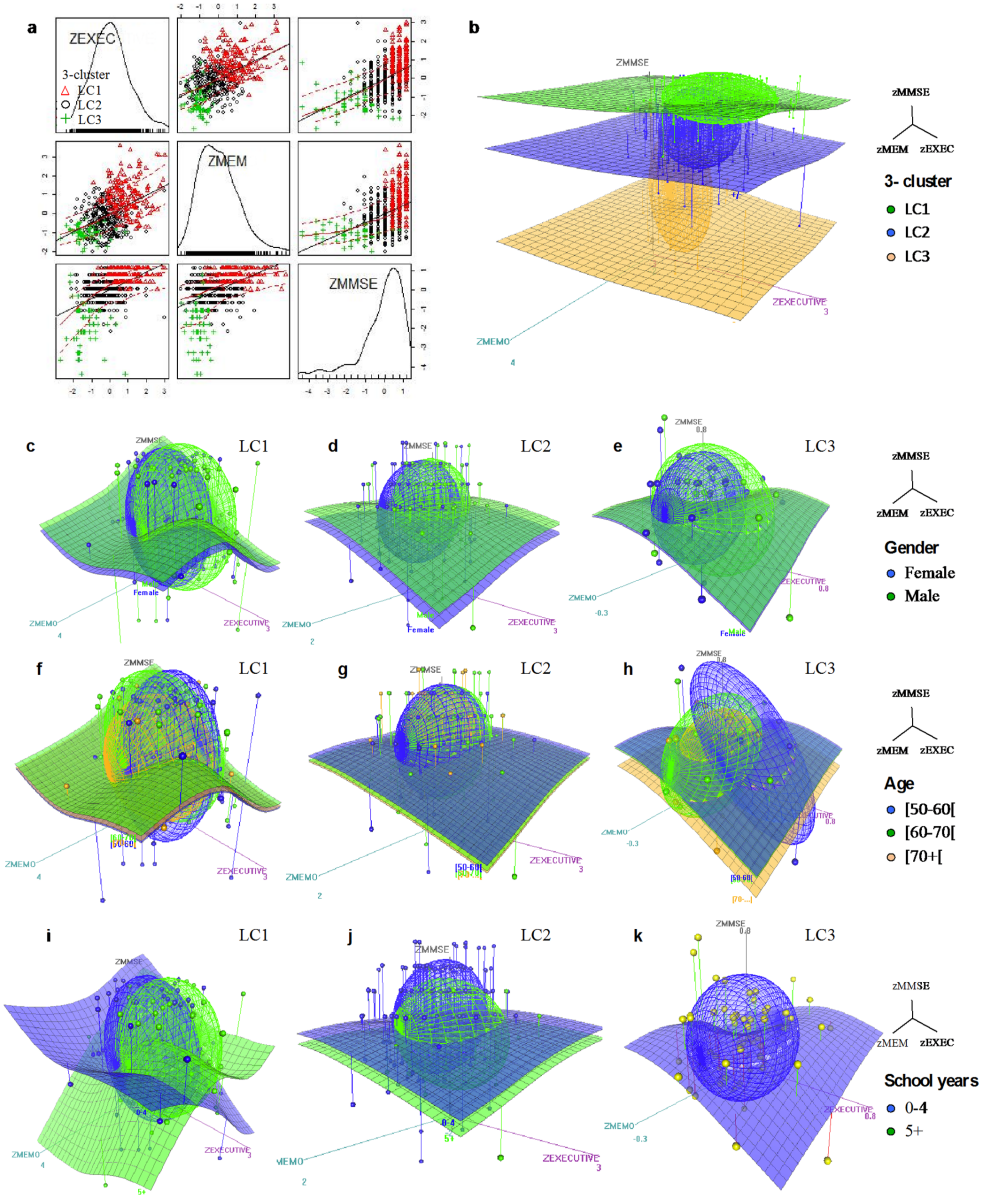
Scatter plot and 3D plots of performance across cognitive dimensions for each latent class (LC1 to LC3) for the 3-cluster Bayesian LCA solution. **a** scatter plot, **b** 3D plot according to latent class, and for each latent class 3D plots regarding **c–e** gender, **f–h** age and **i–k** school years.

The 3-cluster Bayesian LCA solution was considered the strongest: i) had the highest general eta-squared effect, ii) explained ≥5% of the dependent variables compared to the previous applied solution, iii) had a greater variance than that of the previous solution and iv) subjects were evenly distributed among the latent classes. Specifically, the latent classes LC1-LC3 characterized distinct patterns of cognitive performance; 3 in 10 subjects were “strong” performers (LC1), 6 in 10 subjects “average” (LC2), and 1 in 10 “weak” cognitive performers (LC3) for the considered cognitive dimensions (general cognition and executive and memory functions). Scatter plot visualization showed that positive correlations existed between the *MMSE*, *MEM* and *EXEC* dimensions (Fig. 3a). Furthermore, following the cognitive performance pattern (LC1>LC2>LC3, visually represented in the 3D plot Fig. 3b) each latent class overall denoted the same hierarchy in the proportion of males, youth and number of school years across the *MMSE*, *MEM* and *EXEC* dimensions (Fig. 3c–k). MANOVAS were performed in order to detect gender, age and school years main effects in the three considered dimensions split by latent class. Based on Pillai’s Trace gender showed a significant effect on LC1 [F_(3,144)_ = 4.11, p = 0.008, η^2^_p_ = 0.068, π = 0.981; significant differences detected on EXEC] and LC2 [F_(3,295)_ = 7.13, p<0.001, η^2^_p_ = 0.079, π = 0.840; significant differences detected on MMSE and MEM] and not on LC3 [F_(3,52)_<1, p = 0.555, η^2^_p_ = 0.039, π = 0.189]. Significant main effects of age were detected on LC2 [F_(6,590)_ = 4.67, p<0.001, η^2^_p_ = 0.05, π = 0.989; significant differences detected on MEM and EXEC] and on LC3 [F_(6,104)_ = 3.20, p<0.001, η^2^_p_ = 0.16, π = 0.912; significant differences detected on EXEC] and not on LC1 [F_(6,288)_<1, p = 0.857, η^2^_p_ = 0.01, π = 0.178]. Regarding school years significant main effects of were found on LC1 [F_(3,295)_ = 5.86, p = 0.001, η^2^_p_ = 0.06, π = 0.952; significant differences detected on EXEC] and LC2 [F_(3,144)_ = 5.54, p = .001, η^2^_p_ = 0.10, π = 0.937; significant differences detected on MMSE and EXEC]; this analysis was not performed for LC3 because all participants had 0–4 school years.

## Discussion

Latent class analysis is a powerful method for analyzing the relationships among manifest data when some variables are unobserved; variables can represent nominal, ordinal, continuous and count data [4]–[6], [19]. Conceptually, LCA follows other clustering approaches: the original dataset is divided into a number of exclusive and exhaustive subsets (in LCA termed latent classes); however, it uses statistical (rather than mathematical) methodology to construct the results. That is, as opposed to more traditional approaches (e.g. hierarchical or K-means cluster analysis, which we have previously used in our ageing studies [7], [9]), LCA is a model-based approach that offers a probability-based classification through a posterior probability of membership (meaning: cases are not absolutely assigned to classes, but have a probability of membership for each latent class) [6], [19], [20]. Furthermore, LCA models add in some key methodological elements: i) the use of starting values to ensure that the best solution has been found (particularly relevant in complex models with large numbers of classes); ii) does not rely on traditional modeling assumptions (e.g. linear relationship, normal distribution, homogeneity) and is therefore less prone to biases; and iii) the relationship between the latent classes and external variables (covariates) can be assessed simultaneously [5]. In LCA the Expectation Maximization algorithm can be used to obtain the parameters of latent class models; where, in a Bayesian approach, the parameters are usually estimated by Markov Chain Monte Carlo methods. Classic and Bayesian statistical approaches are applied in as multiple and diverse areas as diagnostic testing (for example, [21]) and whole-genome association analyses (for example, [22]), to the modeling of performance typologies (for example, [23]) and clinical studies (for example, [24]–[26]). Particularly, the sociology and psychology fields offer valuable methodological developments regarding LCA models [27]. On ageing research, the approach has been mainly used in longitudinal studies because LCA captures trajectories over long periods of time and can model timing, onset, decline and recovery efficiently in one model.

Herein we used a combined approach of PCA and Bayesian LCA modeling to a cross-sectional analysis in order to identify main cognitive dimensions and to explore patterns of cognitive performance among community-dwelling older individuals. Based on neurocognitive test variables, three main cognitive dimensions were identified that represented general (*MMSE*), memory (*MEM*) and executive (*EXEC*) functions. While the first dimension was a single-variable category, the latter two were composites of multiple test variables; altogether, dimensions were positively correlated. Even noting that the dimensions that were the result of neurocognitive variables groupings (*MEM* and *EXEC*) may be more reliable and/or suitable measures than a cognitive dimension comprised of a single test variable (*MMSE*), here MMSE remained in the analysis as a indicator of general ability. The MMSE is still the most commonly used instrument for screening general cognitive function [28], presenting good reliability indexes, and being an adequate and comparable measure within studies. Nonetheless, some of its limitations have been identified, namely, in detecting for subtle memory losses, particularly in well-educated participants [29], since it is a measure vulnerable to culture and education. In this way, it is possible for a participant to score poorly in the absence of cognitive impairment (for example due to low educational background) or, that higher-educated individuals score well despite having cognitive impairment [30], [31]. In the present study, since the majority of participants had 4 years of school education and were culturally similar, these vulnerabilities most likely did not affect the results or contributed to a possible “ceiling effect.”

From the obtained dimensions, Bayesian LCA was conducted to identify clusters of distinct cognitive performers in the cohort. Three latent classes were identified – LC1 to LC3– which corresponded to stronger to weaker performance across all dimensions (LC1>LC2>LC3). Overall, each latent class denoted the same performance hierarchy in the proportion of males, younger individuals and higher number of school years. Gender, age and school years had the greater impact on LC2 membership; although, it should be noted LC3 membership was only individuals with ≤4 school years. Across each cognitive dimension, gender, age and school years also had an effect on latent class distribution: executive function appeared the most affected by gender (LC1), age (LC2 and LC3) and school years (LC1 and LC2), followed equally by MEM (gender and age, LC2) and MMSE (gender and school years, LC2).

In accordance with our results, two main cognitive domains emerge across studies showing age-related decline: memory and executive function [32]. From a neurocognitive standpoint each of these include a myriad of cognitive processes, where the line between these can be at times difficult to distinguish, particularly as measured by neuropsychological batteries. For instance, memory, while broadly defined as the ability to store and retrieve information (short- and long-term), it can also encompass, for instance, working memory. This capacity implies the ability to transform, re-organize and/or use new information based on previously acquired one. As such, for example, the neurocognitive measure digit-span backward is still debated in the field regarding if it is a measure of short-term or working memory, with some authors indicating that the set of mechanisms used (short-term vs. working) may differ depending on the age group considered [32]. In fact, studies on executive function reveal an even larger group of cognitive processes, ranging from ability to retrieve, maintain and manipulate information, to inhibition, goal switching and formation of new strategies to complete a certain task. Still, in common, memory and executive order function processes show a degree of age-related decline, even if non-uniform inter- or intra-individually throughout ageing [1], [33]. Further interesting, it is thought that some components of both domains can actually be protected from ageing, by “leaning in” on other mechanisms or by process of (repeated) learning [33]. Here, the cross-sectional design does not allow for fine-discrimination among more stable and less stable measures, but it is nonetheless of note that memory and executive function appeared the most affected by age in the weaker classes (LC2 and LC3), possibly indicating that higher performers may rely on a more complex cognitive network.

On this, education plays a major role. In fact, in our previous studies the number of school years was one of the most significant discriminatory parameters in explaining cognitive performance [7], [9], following other similar findings [34], [35]. Here, executive function appeared the most affected by school years particularly in the first two classes (LC1 and LC2). One of the most well-established proxy measures of reserve capacity in older individuals is education, which is thought to promote more efficient cognitive processing and use of brain networks, possibly due to an increase in neural connections made while learning [36], [37]. This means that learning may increase the cognitive reserve, possibly resulting into smaller age-related decline [38] and in relying less on memory strategies to solve problems. Still, interpretation of the results should be taken with care: individuals in the lower educational group are already in a lower cognitive class (“flooring effect”). As such, they have “less to loose,” which may translate into higher educated individuals appearing more “sensitive” to the effects of ageing, albeit this may occur later on [9].

On gender, although differences in cognition have been well documented (for example, women perform better in verbal tasks and men in spatial tasks [39]), differences in ageing effect have not been clearly established across the lifespan. Here, executive function appeared the most affected by gender in the stronger class and memory in the middle class; where, in both cases, males were the stronger performers. While some authors indicate that overall age-decline begins earlier in women than men [40], others defend the opposite [39]. According to Schmand and colleagues, the question may reside on cultural aspects [41]. Older women with a more limited formal education may have poorer cognitive performance compared with men, not because of gender itself, but because of their educational background (which relates to the brain reserve capacity theory). However, this is a matter of debate, particularly for the oldest old. For instance, van Exel and colleagues showed in their cohort study that despite women presenting lower formal education they performed cognitively better than men in the same age class [40]. The authors concluded that formal educational by itself does not explain differences in cognitive performance between genders and, consequently, that the brain reserve theory alone does not explain the gender differences in cognitive performance. Instead, the authors indicated that a later onset of age-associated pathologies, such as cardiovascular disease, could be at the root of the better cognitive functioning of older women compared with men. On this, neurophysiologic and brain structural aspects should also be taken into consideration. Pre-clinical evidence indicates that estrogens have salutary neurophysiologic effects; particularly, in that a drop in estrogen may be detrimental to cognition [42]. So, when considering the mechanisms by which estrogens might impact cognitive function, it is important to consider both indirect (menopausal symptoms such as mood, sleep disturbance, and “hot flashes”) and direct effects (effects on neural systems subserving cognitive functions of hormonal changes on the brain systems) [42]. Another important consideration is that the hippocampus and prefrontal cortex, which serve episodic and working memory, are rich in estrogen receptors [42], which may possibly also underlie gender-specific findings. Altogether, although the findings appear contradictory, it may simply mean that different factors are of different relevance depending on the life stage. Ultimately, if considering that age-associated cognitive performance may rely on compensatory mechanisms [43], poorer performance may relate with a continued reliance on strategies (perhaps verbal in women [39]) that may prove ineffective “leaning-ins” to compensate for the observed age-related decline in more executive and memory-related dimensions.

Some methodological considerations should be taken into account when reporting on the results from the LCA methodology: first, it did not result in the same number of clusters compared to the more traditional procedures (hierarchical and K-means clustering) that we used in our similar datasets (although these with not as many variables and with a smaller sample size without missing values, [7], [9]). Whereas the previous non-latent class analysis strategies yielded 4-clusters, here, LCA yielded a 3-cluster solution; this observation indicates differences in cluster membership between the methods, which is in accordance to the results from Eshghi et al [44] when directly comparing clustering methods using a same database. The apparent inconsistency may be difficult to interpret (particularly when in cases where there are no predefined clusters) and although it may indicate that classical solutions yield more homogeneous clusters, herein the analysis showed that further divisions yielded new classes whose membership performance did not agree with its preceding class. Altogether, although a deeper understanding of different cluster methodologies and their applicability in ageing (cross-sectional) studies is warranted, Bayesian LCA proved to be a powerful technique to inform on cognitive typologies. As complementary tools, logistic regressions or discriminant analysis may prove valuable so that, based on the identified cluster solutions, new subjects are assigned class membership. Finally, strategies of data imputation for missing values (on neurocognitive test variables) may be pertinent as often studies are constrained by these with a considerable impact on sample size and therefore on the use of more powerful statistical techniques.

## Supporting Information

Figure S1 **Frequency polygon of the distribution of the proportion across the 55,500 samples for each Bayesian LCA solution. a, b** 1-cluster (latent classes, LC1 and LC2, respectively), **c–e** 2-cluster (LC1 to LC3, respectively), **f–i** 4-cluster (LC1 to LC4, respectively).
(TIF)
