# Supplementary figures and images for: The Use of Bayesian Latent Class Cluster Models to Classify Patterns of Cognitive Performance in Healthy Ageing
Source: PLoS One. 2013 Aug 20;8(8):e71940. doi: 10.1371/journal.pone.0071940 (PMC3748115; doi:10.1371/journal.pone.0071940)

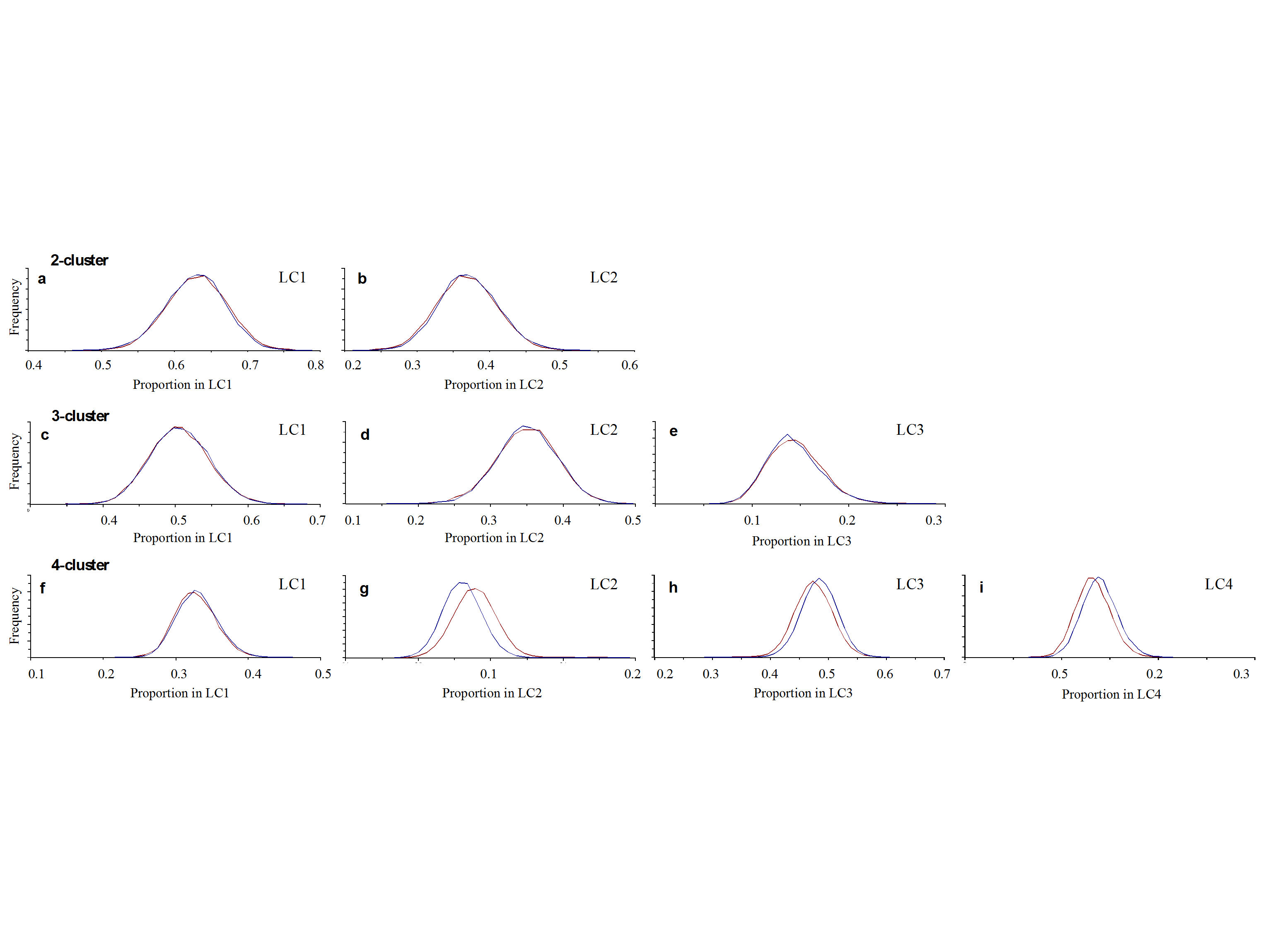

Supplement: Figure S1 — Frequency polygon of the distribution of the proportion across the 55,500 samples for each Bayesian LCA solution. a, b 1-cluster (latent classes, LC1 and LC2, respectively), c–e 2-cluster (LC1 to LC3, respectively), f–i 4-cluster (LC1 to LC4, respectively). (TIF) [file pone.0071940.s001.tif]
